# Physical characteristics of the back are not predictive of low back pain in healthy workers: A prospective study

**DOI:** 10.1186/1471-2474-10-2

**Published:** 2009-01-05

**Authors:** An Van Nieuwenhuyse, Geert Crombez, Alex Burdorf, Geert Verbeke, Raphael Masschelein, Guido Moens, Philippe Mairiaux, the BelCoBack Study Group

**Affiliations:** 1 Department of Epidemiology, Scientific Institute of Public Health, Brussels, Belgium; 2 Department of Public Health, Section of Occupational, Environmental and Insurance Medicine, Katholieke Universiteit Leuven, Leuven, Belgium; 3 Department of Psychology, University of Ghent, Ghent, Belgium; 4 Department of Public Health, Erasmus MC, University Medical Center Rotterdam, Rotterdam, The Netherlands; 5 Department of Public Health, Biostatistical Centre, Katholieke Universiteit Leuven, Leuven, Belgium; 6 External Service for Prevention and Protection at Work IDEWE, Leuven, Belgium; 7 Occupational Health and Health Education unit, Department of Public Health, University of Liège, Liège, Belgium

## Abstract

**Background:**

In the working population, back disorders are an important reason for sick leave and permanent work inability. In the context of fitting the job to the worker, one of the primary tasks of the occupational health physician is to evaluate the balance between work-related and individual variables. Since this evaluation of work capacity often consists of a physical examination of the back, the objective of this study was to investigate whether a physical examination of the low back, which is routinely performed in occupational medicine, predicts the development of low back pain (LBP).

**Methods:**

This study is part of the Belgian Low Back Cohort (BelCoBack) Study, a prospective study to identify risk factors for the development of low back disorders in occupational settings. The study population for this paper were 692 young healthcare or distribution workers (mean age of 26 years) with no or limited back antecedents in the year before inclusion. At baseline, these workers underwent a standardised physical examination of the low back. One year later, they completed a questionnaire on the occurrence of LBP and some of its characteristics. To study the respective role of predictors at baseline on the occurrence of LBP, we opted for Cox regression with a constant risk period. Analyses were performed separately for workers without any back antecedents in the year before inclusion ('asymptomatic' workers) and for workers with limited back antecedents in the year before inclusion ('mildly symptomatic' workers).

**Results:**

In the group of 'asymptomatic' workers, obese workers showed a more than twofold-increased risk on the development of LBP as compared to non-obese colleagues (RR 2.57, 95%CI: 1.09 – 6.09). In the group of 'mildly symptomatic' workers, the self-reports of pain before the examination turned out to be most predictive (RR 3.89, 95%CI: 1.20 – 12.64).

**Conclusion:**

This study showed that, in a population of young workers wh no or limited antecedents of LBP at baseline, physical examinations, as routinely assessed in occupational medicine, are not useful to predict workers at risk for the development of back disorders one year later.

## Background

Low back pain (LBP) is a prevalent health problem that imposes an enormous burden on individuals and society [1]. Work-related factors such as bending and twisting, whole-body vibration, manual materials handling and individual variables such as history of pain and age have been consistently associated with the occurrence of low back pain in diverse settings, including occupational ones [2]. At the work place, prevention has therefore focused upon the elimination of risk factors associated with physical load. Another strategy is to identify those employees at risk for developing low back pain, and to adjust work-related capacity as a function of the individual work capacity [3,4]. Such an analysis is accomplished through a physical examination by an occupational health physician.

The evaluation of work capacity by an occupational health physician often consists of a physical examination of the back. According to a biomechanical vision, low back pain is caused by musculoskeletal dysfunctions at the level of the motion segments [5]. Musculoskeletal dysfunctions, for example, could be assessed by evaluating the following physical characteristics of the back [6,7]: (1) the static aspects such as kyphosis, lordosis, iliac crest inequality and scoliosis, (2) the spinal flexibility, (3) muscle force, (4) pain by palpation, and (5) a neurological assessment of the lower extremities. Body constitution is also often considered. An increased body mass index has been suggested to be associated with LBP by 1) excessive wear and tear resulting from the increased mechanical demands and/or 2) by detrimental metabolic factors associated with obesity [8]. More research about the validity of such a physical examination in predicting low back episodes in working populations is however warranted [9-19]. First, there are only a few prospective studies that have investigated the predictive value of a physical examination in working populations, and the results of these studies are mixed. There is evidence that a limitation of the range during a straight-leg raising test is predictive of future low back pain [18,19]. Also the experience of back pain during the physical examination is predictive of future pain [10,17-19]. Second, most studies have investigated the validity in workers who already complain about LBP, making it difficult to infer whether the results of the physical examination are due to real physical abnormalities or due to pain increases during the examination. Such design leaves the question unanswered whether future low back pain may be predicted by the same physical characteristics. The objective of this study is then (1) to assess the value of individual physical characteristics in predicting future low back pain in a sample of young workers without any antecedents of back disorders, (2) to assess the value of individual physical characteristics in predicting future low back pain in a sample of young workers with limited antecedents of back disorders, and (3) to compare the results between these two groups.

## Methods

### 1. Subjects

This study is part of the Belgian Low Back Cohort (BelCoBack) Study, a prospective study to identify risk factors for the development of low back disorders in occupational settings. To this purpose, we included only workers aged 30 years or less and with no or limited antecedents of LBP, *i.e*. less than one week consecutively in the year before intake. To minimise drop-out, workers had to have a tenured position or equivalent. Participants were recruited among the employees of four health care institutions and two distribution companies throughout Belgium. These sectors of employment were regarded to at risk for an elevated occurrence of LBP. The recruitment took place as a result of the annual medical examination by the occupational health physician. In Belgium, such an examination is obliged by law for workers exposed to occupational risks [20].

At baseline, physical and psychosocial workload and individual characteristics were assessed by questionnaires and a physical examination of the low back. One year later, workers filled in another questionnaire. In this questionnaire, we registered the occurrence of low back pain lasting one week or more consecutively in the follow-up year as well as some consequences.

In total, 1672 employees aged 30 years or less and with a tenured position or equivalent were contacted for the study. Twelve-hundred employees (72%) agreed to participate. However, during a first contact, 159 were excluded because they did not meet the inclusion criterion of no or limited antecedents of LBP in the year before intake (*i.e*. they had suffered from LBP during a week or more consecutively in the year before intake), leaving a sample of 1041 workers.

Of those 1041 workers, 972 (93%) completed the questionnaire at baseline. A physical examination of the low back at baseline was carried out in the majority of these 972 workers (n = 942, 97%). Out of the 942 workers with both questionnaire and physical examination at baseline, 776 (82%) returned the questionnaire one year later. The results of the questionnaires have been reported earlier [21] for a subcohort of workers. In that article, we investigated the effect of work-related factors and individual characteristics on the occurrence of LBP. To this purpose, a subcohort of 851 workers with a minimal experience of at least two months in their function at intake was identified. An interval of two months was considered sufficient to appreciate the work constraints in a function. The majority of these 851 workers (n = 823, 97%) had had a physical examination of the low back at baseline. Of the 823 workers with both questionnaire and physical examination at baseline, 692 (84%) sent back the questionnaire one year later.

The mean age in these 692 workers was 26 years (SD of three years), 60% were women. Sixty-three percent were recruited in health care institutions and thirty-seven percent in distribution companies. Despite our efforts to select a population with minimal back complaints, 49% reported back complaints lasting longer than one day in the year before intake. However, due to the inclusion criteria, these complaints were limited to less than one week.

The study protocol was approved by the local Commission for Medical Ethics, and an informed consent was obtained from each employee prior to participation in the study.

### 2. Data collection

Physical examination of the low back consisted of tests that are routinely used in occupational medicine [22]. It was conducted by two occupational health physicians (EE, GVR) and three research assistants (AVN, DP, AL). 80% of the examinations were carried out by the research assistants. Examiners were intensively trained. Total duration of the examination was about 15 minutes. The order of tests was similar for all participants. During the physical examination, both physical measures and self-reports of pain during tests were obtained. Height and weight were reported in the questionnaire. Before the start of the examination, participants were also asked whether they currently experienced LBP. The different items of the protocol are summed up in Additional file 1.

Inter-examiner repeatability of the physical examination was calculated in a pilot study [23]. Briefly summarised, AVN and DP independently examined 30 volunteers; AVN and AL examined another 30 volunteers. These volunteers were not recruited from the final study sample. They were mainly students, but were -like the participants in the BelCoBack study- not older than 30 years at the time of examination and had not suffered from LBP lasting one week or more consecutively in the year before examination. They received a movie ticket for participation. A written informed consent was obtained. The same registration form as in the study was used. The inter-examiner repeatability for categorical variables resulted in kappa values ranging from 0.35 to 1.00 indicating fair to perfect agreement [24]. For the continuous variables, we obtained intraclass correlation coefficients (ICC's) ranging from 0.81–0.94 for continuous variables, except for lumbar extension with an ICC of 0.54. Thus, the physical examination showed acceptable agreement between examiners, except for the range of lumbar extension. Therefore, the range of lumbar extension was not taken into account in the further analyses.

### 3. Statistical analysis

Outcome of the statistical analyses was the development of LBP lasting one week or more consecutively after one year of follow-up. For the analyses, the study population was divided in two subgroups: workers without any antecedents in the year before intake ('asymptomatic' workers) and workers with limited antecedents in the year before intake ('mildly symptomatic' workers). This last group comprised thus the workers who reported back complaints lasting longer than one day, but less than one week consecutively in the year before intake.

The impact of individual factors on the incidence risk was first studied by univariate analyses. For categorical data, we used Chi-square tests or Fisher-Exact tests and calculated relative risks (with 95% confidence intervals). For continuous data, we applied Mann Whitney U or unpaired T-tests. Univariate analyses were carried out for the different individual factors separately as well as for a combined variable. This combined variable was created *a posteriori *because of the low prevalence of abnormal scores on each of the individual factors and consequently the low power to show an effect. It was constructed by a combination of the different individual factors: workers that scored 'abnormal' in at least one item were scored 'abnormal' in the combined variable; workers that scored 'normal' in all items were categorised as 'normal' in the combined variable. Pain elicited in the straight-leg-raising test was not included because nobody complained of sciatic pain. As the study population was rather specific (young workers with no or limited antecedents of LBP), we could not rely on reference values for the categorisation of the continuous variables. Therefore, we decided to classify scores above P90 as 'abnormal' and to use this categorisation for the construction of the combined variable.

Second, we performed multivariate analyses. Classical logistic regression yields odds ratios as measures for the association between prognostic factors and the response of interest. In order to obtain relative risks, we opted for Cox regression with a constant period of risk for all participants [25]. Variables that met a 20% level of significance in the univariate analyses were considered for inclusion in the multivariate analyses. We calculated correlation coefficients among these variables as an approximate manner to detect collinearity. Age and gender were included irrespective of their relation with LBP. In the final multivariate models only variables with a P level less than 0.05 were retained; non-significant variables were removed by means of a backward selection procedure. All analyses were conducted with the SPSS computer package (version 10).

## Results

### 1. Descriptive statistics of the outcome

During the one year of follow-up, 12.5% (95%CI: 10.0–14.9) of the 692 workers developed LBP lasting one week or more consecutively. This incidence did not differ significantly between men (11.9%, 95%CI: 8.1 – 15.7) and women (12.9%, 95%CI: 9.6 – 16.0).

It did differ, however, between the 'asymptomatic' and the 'mildly symptomatic' workers. The latter group consisted of 337 workers of whom 66% were women, the former group comprised 355 workers including 54% women. The mean age and standard deviation were 26 +/- 3 years in both groups. In the group of the 'asymptomatic' workers, 9.6% (95%CI: 6.5 – 12.6) reported the occurrence of LBP lasting one week or more consecutively during follow-up, whereas in the group of the 'mildly symptomatic' workers, this proportion increased to 15.5% (95%CI: 11.6 – 19.3).

### 2. Individual risk factors for low back pain

#### 2.1. Descriptive statistics

The prevalence of abnormalities at baseline was low for most of the items: before the physical examination, 1.2% of the 'mildly symptomatic' workers reported current LBP. Pain reporting during the examination was also limited: of the 'asymptomatic workers', 2.3% reported pain in the low back or buttock in flexion, 0.6% in lateral flexion to the right and 0% in lateral flexion to the left. For the 'mildly symptomatic' workers, these percentages were 0.9%, 3.0% and 2.7%, respectively. Pain in the low back in passive extension was elicited in 2.9% of the 'asymptomatic' and 7.8% of the 'mildly symptomatic' workers. The neurological examination scored abnormal for 2.0% of the 'asymptomatic' workers at the right side and for 1.1% at the left side, and for 1.8% of the 'mildly symptomatic' workers at the right side and for 2.4% at the left side.

For only three items, the baseline prevalence was higher. In 4.8% of the 'asymptomatic' and 3.6% of the 'mildly symptomatic' workers, an iliac crest height inequality of at least 1.5 cm was observed. Scoliosis was registered in 15.5% ('asymptomatic') and 13.4% ('mildly symptomatic') of the workers. 71.8% of the 'asymptomatic' and 64.1% of the 'mildly symptomatic' workers reported pain in the straight-leg raising test right. The straight-leg raising test left was painful in 69.2% of the 'asymptomatic' and 63.2% of the 'mildly symptomatic' workers. It is noteworthy that only local pain (low back, buttock, thigh or knee, other places) was found. Nobody complained of radiating pain during the straight-leg raising test.

Due to the low prevalence for most of the items, the categorical items that were measured both on the right and the left side were recoded as one item (right or left) and treated as such in the further analyses.

#### 2.2. Univariate analyses

##### 2.2.1. Univariate analyses for the separate individual factors

Tables 1 and 2 present the univariate analyses for the categorical and continuous variables. Analyses are presented both for the 'asymptomatic' and for the 'mildly symptomatic' workers.

**Table 1:** Categorical risk factors for LBP lasting seven or more consecutive days after one year of follow-up (LBP at t1) in univariate analyses.

		**Asymptomatic workers**						**Mildly symptomatic workers**			
			LBP at t1					LBP at t1			
		n	%	RR	95%CI	P-value	n	%	RR	95%CI	P-value
Current LPB (reported before the physical examination)	No	354	9.6	/	/	/	331	14.8	1.00		**0.013**
	Yes	/	/				4	75.0	**5.07**	**(2.72 – 9.44)**	

Iliac crest height inequality ≥ 1.5 cm	No	336	9.5	1.00		0.673	320	15.6	1.00		0.700
	Yes	17	11.8	1.23	(0.32 – 4.74)		12	8.3	0.53	(0.08 – 3.54)	

Scoliosis	No	299	10.0	1.00		0.523	289	16.3	1.00		0.375
	Yes	55	7.3	0.72	(0.27 – 1.98)		45	11.1	0.68	(0.29 – 1.63)	

Lumbar flexion: Pain in the low back or buttock	No	343	9.6	/	/	1.000	330	15.5	1.00		0.400
	Yes	8	0.0				3	33.3	2.16	(0.43 – 10.90)	

Lumbar extension: Pain in the low back	No	333	9.6	/	/	0.608	305	13.4	1.00		**0.008**
	Yes	10	0.0				26	34.6	**2.58**	**(1.41 – 4.69)**	

Pain in the low back or buttock in lateral flexion	No	349	9.7	/	/	1.000	325	14.5	1.00		**0.010**
	Yes	2	0.0				10	50.0	**3.46**	**(1.76 – 6.80)**	

Pain in the low back or buttock-thigh or knee- other places in SLR	No	99	9.1	1.00		0.838	118	16.9	1.00		0.595
	Yes	255	9.8	1.08	(0.52 – 2.23)		217	14.7	0.87	(0.52 – 1.45)	

Neurological examination of the lower limbs	Normal	345	9.3	1.00		0.548	327	15.6	1.00		1.000
	Abnormal	8	12.5	1.35	(0.21 – 8.70)		8	12.5	0.80	(0.13 – 5.10)	

Body mass index (kg/m^2^)	Normal	233	8.6	1.00		**0.026**	235	14.5	1.00		0.359
	Overweight	65	6.2	0.72	(0.25 – 2.02)		64	21.9	1.51	(0.87 – 2.64)	
	(≥ 25 and <30)										
	Obese (>30)	31	22.6	**2.63**	**(1.21 – 5.71)**		18	16.7	1.15	(0.39 – 3.39)	

P value calculated with Chi-square or Fisher-Exact tests/RR = relative risk/95%CI = 95% confidence interval

**Table 2:** Continuous risk factors for LBP lasting seven or more consecutive days after one year of follow-up (LBP at t1) in univariate analyses.

	**Asymptomatic workers**					**Mildly symptomatic workers**				
	LBP at t1		No LBP at t1		P-value	LBP at t1		No LBP at t1		P-value
	n	Median (Q1-Q3)	n	Median (Q1-Q3)		n	Median (Q1-Q3)	n	Median (Q1-Q3)	
Fingertip-to-floor distance (cm)	34	0.0 (0.0–0.0)	318	0.0 (0.0–5.3)	0.094	52	0.0 (0.0 – 5.5)	283	0.0 (0.0–3.3)	0.524

Difference in excursion of the middle finger on the thigh between lateral flexion to the right and to the left (cm)	34	1.3 (0.5 – 3.0)	318	1.5 (1.0 – 3.0)	0.818	52	1.0 (0.0 – 2.0)	283	2.0 (0.5 – 3.0)	**0.003**

Difference between the range in straight-leg-raising right -left (degrees)	32	5.0 (0.0 – 10.0)	318	5.0 (0.0 – 5.0)	0.259	52	5.0 (0.0 – 5.0)	278	5.0 (0.0 – 5.0)	0.146

Difference between the range of the hamstring muscles right – left (degrees)	31	0.0 (0.0 – 0.0)	311	0.0 (0.0 – 5.0)	0.440	52	0.0 (0.0 – 5.0)	270	0.0 (0.0 – 5.0)	0.799

P-value calculated with Mann Whitney U tests

###### 'Asymptomatic' workers

Obese workers showed an almost threefold increased risk on LBP lasting a week or more consecutively one year later (RR 2.63, 95%CI: 1.21 – 5.71).

###### 'Mildly symptomatic' workers

In the group of the 'mildly symptomatic' workers, both pain reported before examination and reports of pain increases during examination were predictive of LBP one year later. This was the case for current LBP reported before the physical examination, LBP elicited in passive back extension and pain in the low back or buttock provoked in lateral flexion. Pain at the straight-leg raising test was not associated with LBP. It has to be stressed however that nobody complained of sciatic pain. Only local pain, *i.e*. pain in the low back or the buttock, at the thigh or knee, or at other places, was reported. Of those items that evaluated the range of mobility, only the difference in excursion of the middle finger on the thigh between lateral flexion to the right and to the left was significant (Mann Whitney U test, P = 0.003). The median difference in lateral flexion right-left amounted to one cm in workers who developed LBP lasting one week or more one year later and to two cm in workers who did not (Table 2). Thus, the practical significance of this statistical significant finding is very limited. Neither postural abnormalities (iliac crest height inequality-scoliosis) nor the tests that evaluated the neurological function of the lower limbs predicted LBP one year later.

##### 2.2.2. Univariate analyses for the combined variable

Univariate analyses for the combined variable are given in Table 3. As explained in the methods, this variable was constructed *a posteriori *because of the low prevalence of positive scores on each item separately. Analyses are presented for the 'asymptomatic' and the 'symptomatic' workers.

**Table 3:** Risk (combined variable) for LBP lasting seven or more consecutive days after one year of follow-up (LBP at t1) in univariate analyses.

		**Asymptomatic workers**					**Mildly symptomatic workers**				
			LBP at t1					LBP at t1			
		n	%	RR	95%CI	P-value	n	%	RR	95%CI	P-value
Combined variable	Normal	156	7.7	1.00		0.591	173	15.6	1.00		
	Abnormal	171	9.4	1.22	(0.60 – 2.49)		146	15.8	1.01	(0.61 – 1.68)	0.971

P value calculated with Chi-square tests/RR = relative risk/95%CI = 95% confidence interval

###### 'Asymptomatic' workers

A deviant result on at least one item of the physical examination was found in 52.1% of the workers. Workers with at least one deviant result showed a 1.22-fold increased risk on LBP one year later as compared to workers who scored normal on all the items of the physical examination. However, the result was not significant (95%CI: 0.60 – 2.49, Chi-square test: P = 0.591).

###### 'Mildly symptomatic' workers

In the group of the 'mildly symptomatic' workers, 46.1% scored deviant in at least one item of the physical examination. The risk on LBP for workers with any deviant score versus their colleagues with overall normal scores was 1.01, and not significant (95%CI: 0.61 – 1.68, Chi-square: P = 0.971).

#### 2.3. Multivariate analyses

Variables associated with a P value ≤ 0.20 in univariate analyses were considered for inclusion in the multivariate analyses. Age and gender were included as epidemiological confounders, irrespective of their relationship with LBP. Results for the 'asymptomatic' and 'mildly symptomatic' workers are shown in Table 4.

**Table 4:** Risk factors for LBP lasting seven or more consecutive days after one year of follow-up (LBP at t1) in multivariate analyses.

**Asymptomatic workers**^**a**^										
			LBP at t1		UNIVARIATE ANALYSES			MULTIVARIATE ANALYSES		
		n	n	%	RR	95%CI	P-value	RR	95%CI	P-value
Body mass index (kg/m^2^)	Normal	228	20	8.8	1.00		0.056	1.00		0.056
	Overweight	63	4	6.3	0.72	(0.25 – 2.12)		0.72	(0.25 – 2.12)	
	(≥25 and <30)									
	Obese (≥ 30)	31	7	22.6	**2.57**	**(1.09–6.09)**		**2.57**	**(1.09 – 6.09)**	

**Mildly symptomatic workers **^**b**^										
Current LBP (reported before the physical examination)	No	322	49	15.2	1.00		**0.007**	1.00		**0.024**
	Yes	4	3	75.0	**4.93**	**(1.54–15.81)**		**3.89**	**(1.20 – 12.64)**	
Difference in excursion of the middle finger on the thigh between lateral flexion to the right and to the left (cm)										
					1.00		**0.014**	1.00		**0.023**
					**0.77**	**(0.62–0.95)**		**0.78**	**(0.63–0.97)**	

RR = relative risk/95%CI = 95% confidence interval

^a ^Cox-regression, backward selection, p_in _= 0.20, p_out _= 0.05. Adjusted additionally for the fingertip-to-floor distance, age and gender.

^b ^Cox-regression, backward selection, p_in _= 0.20, p_out _= 0.05. Adjusted additionally for the difference between the range of straight-leg-raising right-left (degrees), age and gender.

##### 'Asymptomatic' workers

In the multivariate analyses, none of the individual factors was significantly related to low back disorders one year later. As compared to their colleagues with normal body mass index, the group of obese workers showed a more than twofold increased risk on LBP lasting one week or more consecutively one year later (RR 2.57, 95%CI: 1.09 – 6.09). As can be seen from table 4, however, the variable 'body mass index' as a whole was borderline not significantly predictive for LBP one year later (overall P-value 0.056).

##### 'Mildly symptomatic' workers

Of the three pain indicators, *i.e*. 'current LBP reported before the physical examination' 'LBP elicited in passive back extension', and 'pain in the low back or buttock provoked in lateral flexion', only the first two remained significant in multivariate analyses. However, the level of significance lowered to a great extent due the inclusion of the different pain indicators. It was therefore considered wise to include only the most significant pain indicator in the final model building, *i.e*. 'current LBP reported before the physical examination'. The final model yielded elevated risks for (1) current LBP reported before the physical examination, and (2) the difference in excursion of the middle finger on the thigh between lateral flexion to the right and to the left.

## Discussion

This study investigated the predictive value of a physical examination, which is routinely applied in occupational medicine, in a sample of young workers with no or only limited antecedents of back disorders. The results can be readily summarized. Firstly, almost one out of eight workers developed low back complaints lasting one week or more in the one year of follow-up. Secondly, overall, tests were reliable, but only few abnormalities were observed. Thirdly, physical items proved not interesting in predicting LBP. Only the report of pain before and during the examination had predictive value.

Of particular interest in this study was the effect of physical variables on the development of LBP. Previous prospective studies have focused on workers with complaints and showed that pain increases during the examination were related to future LBP. To clarify the influence of physical abnormalities in the absence of the so dominant pain characteristics, we selected workers with no or only a limited history of back pain. Workers older than 30 years or with LBP lasting one week or more consecutively in the year before inclusion were excluded. Despite our efforts to select an asymptomatic population, about half of the workers indicated some back complaints, *i.e*. longer than one day but less than one week consecutively, in the year before intake and four of them reported back complaints at the day of the examination. Therefore, in the analyses, we made a distinction between workers without any history of back problems and workers with some, but a limited history in the year before intake.

Individual physical variables proved not useful at all for screening workers with no or limited back antecedents at risk for reporting LBP in the future. First, there were only a limited number of physical abnormalities. Second, none of the physical variables consistently predicted LBP one year later. This was not only the case for the abnormalities with low baseline prevalence (and consequently lower power to reach significance), but also for the abnormalities with higher baseline prevalence. Of the ranges of mobility, only the difference between lateral flexion to the right and to the left was statistically significant in workers with limited antecedents. However, this statistical difference proved of no practical significance. Other authors have also found side to side differences to be predictive for LBP problems. In workers of a Finnish forest industry enterprise [19], a side difference ≥20° in the straight-leg-raising angle predicted sick leave more than 14 days. More specifically, the combination of side difference in straight-leg-raising angle ≥20° and pain below the knee and relief of pain when lying and severe trouble at work turned out to be highly predictive to identify a small subgroup (6%) of the study population at extremely high risk for sick leave more than 14 days. However, the study population, *i.e*. workers visiting the occupational health service for medical advice for low back disorders, was quite different from the 'asymptomatic' and 'mildly symptomatic' population in our study. Nadler and co-workers [26] showed that treatment for LBP in female collegiate athletes was predicted by the percentage difference between the right and left hip extensors. For men, this association was not significant.

In workers without any back history, only obesity was found of importance both in univariate and multivariate analyses. However, the epidemiological evidence for this factor remains unclear in literature [8]. Furthermore, as several tests have been carried out, significant results may be due to multiple testing.

The variables that were most predictive for LBP one year later were the self-reports of pain before the start of the examination and the self-reports of pain provocation during the actual tests. Moreover, the self-report of pain before examination proved more important than any of the reports of pain during examination. Hence, while less time-consuming and intensive than a physical examination, a simple self-report of pain is more informative than a physical examination. Our results are in agreement with those of the Boeing study. In that study, Bigos and co-workers concluded that, once historical information about previous pain (treatment) was known, information gained from physical factors added no significant predictive value [27]. Other than a history of back problems, the authors identified work perceptions and psychosocial factors to be most predictive of future reporting of back injury [28].

The value of self-reports of pain in our population of 'mildly symptomatic' workers however remains limited for the purpose of screening. Of the four workers that reported LBP at baseline, three (75%) developed LBP one year later whereas one (25%) did not. Such a value is not enough for the identification of those at risk, as still 25% of the workers with pain at baseline did not develop LBP one year later. Furthermore, the low prevalence of self-reported pain at baseline (only 4 of the 692 workers reported pain at baseline) raises doubts about the costs-benefits ratio.

Because of the low prevalence of abnormal scores on each of the individual items separately, we have also constructed a combined variable. Workers that scored 'abnormal' in at least one individual item were scored deviant in the combined variable. A 'normal' score for the combined variable was given to the workers who scored 'normal' on all the individual items. By doing so, we increased the number of positive findings and consequently the power to show an effect. However, despite this effort, our physical examination was not significantly related to LBP one year later. This negative result may again be due to lack of power. Power calculations *a posteriori *indicated that, even for the combined variable, we would have needed 4981 workers with deviant scores and 4544 with overall normal scores in the group of the 'asymptomatic' workers to be able to show a significant difference between the proportions found with a power of 90% and alpha = 0.05 one-sided. For the group of the 'mildly' symptomatic workers, this numbers would have amounted to 523 405 workers with any deviant score and 620 148 workers with overall normal scores. Since so many workers are needed to show any effect, this finding stresses once more that, from a cost-benefit point of view, physical examinations as carried out by our protocol are not useful in screening workers with no or limited antecedents at risk for LBP.

Few prospective studies have investigated the predictive value of physical examinations. In most of these studies, subjects underwent a physical examination following the reporting of back disorders. Our study indeed lends support to a relationship between some pain provocation tests and LBP, but neither a limited range in the straight-leg raising tests, nor pain elicited in straight-leg raising was predictive for LBP. We should however be mindful that we had no workers with signs of root compression/inflammation at baseline either.

## Conclusion

We studied the predictive value of individual physical characteristics in a young population with no or only limited antecedents of the back. Physical variables as measured by a standard physical examination proved not predictive for the reporting of LBP one year later. Of interest is that there were not many abnormalities at baseline. Only self-reports of pain were related to LBP one year later.

## Abbreviations

LBP: low back pain; RR: relative risk; 95%CI: 95% confidence interval.

## Competing interests

The authors declare that they have no competing interests.

## Authors' contributions

AVN participated in the conception and design of the study, data collection, data processing and data analysis, and drafted the manuscript. GC participated in the conception and design of the study, the data analysis, and helped to draft the manuscript. AB participated in the conception and design of the study, the data analysis, and helped to draft the manuscript. GV participated in the data analysis, provided statistical advice, and reviewed the manuscript. RM participated in the concept and design of the study, was responsible of the supervision of the data collection, data processing and analyses, and reviewed the paper. GFM conceived the study and its design, was responsible of the supervision of data collection, data processing and analyses, and reviewed the paper. PM participated in the concept and design of the study, was responsible of the supervision of data collection, data processing and analyses, and reviewed the paper.

## Pre-publication history

The pre-publication history for this paper can be accessed here:



## Supplementary Material

Additional file 1 **Appendix** Click here for file
