# Supplementary material for: Physical characteristics of the back are not predictive of low back pain in healthy workers: A prospective study
Source: BMC Musculoskelet Disord. 2009 Jan 5;10:2. doi: 10.1186/1471-2474-10-2 (PMC2630962; doi:10.1186/1471-2474-10-2)
Supplement: Additional file 1 — Appendix [file 1471-2474-10-2-S1.doc]

| **Appendix: Items in the standardised examination protocol for the low back** | | |
| --- | --- | --- |
|  |  |  |
| **Item** | **Measurement instrument** | **Scale/classification** |
|  |  |  |
| **Iliac crest height inequality** [7,12] | Visual assessment and palpation | No/Yes |
|  | Wooden boards of 0.5 cm thickness | Difference Right versus Left in cm |
|  |  | (accuracy of 0.5 cm) |
|  |  |  |
| **Scoliosis** [7] | Visual assessment | Absent / Present |
|  |  |  |
| **Lumbar flexion** [7] |  |  |
| Range | Tape measure | Fingertip-to-floor distance in cm  (accuracy of 0.5 cm) |
|  |  |  |
| Pain in the low back or buttock |  | No/Yes |
|  |  |  |
| **Lumbar extension** [7] |  |  |
| Range | Inclinometer | Difference between extension at T12-L1 and S1 in degrees |
|  |  | (accuracy of 5 degrees) |
|  |  |  |
| Pain in the low back in passive extension |  |  |
|  |  |  |
| **Lateral flexion** [7] |  |  |
| Range | Tape measure | Excursion of middle finger on the thigh in cm Right/Left  (accuracy of 0.5 cm) |
|  |  |  |
| Pain in the low back or buttock |  | No/Yes Right/Left |
|  |  |  |
| **Straight leg raising** [7] |  |  |
| Range | Inclinometer | Elevation angle in degrees Right/Left  (accuracy of 5 degrees) |
|  |  |  |
| Pain: sciatic pain – low back or buttock - |  |  |
| thigh or knee - other |  | No/Yes Right/Left |
|  |  |  |
|  |  |  |

| **Appendix (ct’d): Items in the standardised examination protocol for the low back (ct’d)** | | |
| --- | --- | --- |
|  |  |  |
| **Item** | **Measurement instrument** | **Scale/classification** |
|  |  |  |
| **Length of hamstring muscles** [29] | Inclinometer | Angle in degrees Right/Left |
|  |  | (accuracy of 5 degrees) |
|  |  |  |
| **Peripheral neurological** |  |  |
| **examination of the lower limbs** [7] |  |  |
| Strength testing L4/L5/S1 |  | Norma/Abnormal Right/Left |
|  |  |  |
| Reflexes L4/S1 |  | Present/Absent Right/Left |
|  |  | Symmetric/Asymmetric |
